# Anabolic Actions of the Regenerative Agent Enamel Matrix Derivative (EMD) in Oral Periosteal Fibroblasts and MG 63 Osteoblasts, Modulation by Nicotine and Glutathione in a Redox Environment

**DOI:** 10.3390/jfb3010143

**Published:** 2012-02-29

**Authors:** Tareq Al-Qattan, Mena Soory

**Affiliations:** 1 Periodontology, King’s College London Dental Institute, Guy’s Dental Hospital, London SE1 9RT, UK; E-Mail: tareq.al-qattan@kcl.ac.uk; 2 Periodontology, King’s College London Dental Institute, King’s College Dental Hospital, London SE5 9RW, UK

**Keywords:** enamel matrix derivative, redox status, antioxidant, regeneration

## Abstract

Our study seeks to explore anabolic effects of a periodontal regenerative agent enamel matrix derivative (EMD). Its modulation by nicotine and the anti-oxidant glutathione (GSH) are investigated in human periosteal fibroblasts (HPF) and MG63 osteoblasts. Androgen biomarkers of oxidative stress and healing, resulting from radiolabeled androgen substrates are assayed. This *in vitro* model simulates a redox environment relevant to the periodontal lesion. It aims to confirm the hypothesis that EMD is an effective regenerative agent in a typically redox environment of the periodontal lesion. Monolayer cultures of MG63 osteoblasts and HPF established in culture medium are incubated with androgen substrates, and optimal concentrations of EMD, nicotine and GSH, alone and in combination. EMD significantly enhances yields of 5α-dihydrotestosterone (DHT) an effective bioactive metabolite, alone and in combination with GSH, to overcome oxidative effects of nicotine across cultures. The ‘*in vitro*’ findings of this study could be extrapolated to “*in vivo*” applications of EMD as an adjunctive regenerative therapeutic agent in an environment of chronic inflammation and oxidative stress. Increased yields of DHT implicated in matrix synthesis and direct antioxidant capacity, confirm the potential applications for enamel matrix derivative in periodontal regenerative procedures.

## 1. Introduction

The aim of this *in vitro* investigation is to study anabolic responses to enamel matrix derivative (EMD) in human periosteal fibroblasts and osteoblasts in a redox environment, using radiolabeled steroid substrates. The actions of EMD in an inflammatory environment are relevant to its effects in regenerating periodontal bone defects. The rationale for this investigation is supported by the actions of the agents studied with potential for extrapolation to the *in vivo* environment. Relevant background on agents used in this study and their potential applications are provided here. 

### 1.1. Regenerative Capacity of Enamel Matrix Derivative

The scientific basis for the regenerative actions of EMD and clinical application in periodontal recession defects are documented [1,2]. In addition to its periodontal regenerative role, EMD reduces the incidence of post-operative pain and swelling; and accelerates wound healing, suggestive of an anti-inflammatory role in periodontal healing [3]. Down-regulation of genes associated with the early inflammatory phase of periodontal wound healing is attributed to EMD, with simultaneous up-regulation of genes encoding for molecules that promote growth and repair [4]. 

Others have shown that EMD limits the release of pro-inflammatory cytokines from stimulated blood cells, supporting its anti-inflammatory role [5]. Anti-inflammatory effects of EMD in rat monocytes exposed to bacterial lipopolysaccharide have been reported by others [6]. These studies are indicative of a positive effect of EMD in reducing inflammation by down-regulating pro-inflammatory molecules and advancing healing by reducing its inflammatory component. Heavy occlusal loading could reduce the anti-inflammatory effects of EMD, leading to a less favorable healing response following surgical intervention [7]. Several studies have shown that EMD stimulates attachment of periodontal ligament fibroblasts and enhances alkaline phosphatase (ALP) activity [8,9,10,11], an enzyme associated with the turnover of connective tissue and bone. 

### 1.2. Anabolic and Antioxidant Actions of Androgens

The physiological actions of androgens and estrogen contribute to matrix synthesis and wound healing [12]. In addition, they can be used to overcome tissue catabolism associated with chronic disease, due to their anabolic and immune-modulatory effects [13,14]. Anabolic actions of testosterone and estrogen in restoring extracellular matrix loss have been demonstrated in an inflamed rat model. Administration of physiological levels of DHT and estrogen subcutaneously restored antioxidant status with reduced levels of TNF-α and MMP-2 [15], demonstrating antioxidant and matrix-enhancing actions of DHT. The physiologically active androgen DHT has also been shown to increase ALP activity [12]. This could partly mediate the anabolic influence of androgen metabolites in tissues. Gingival tissue is a target for androgen hormones whereby physiological concentrations of circulating androgens influence the formation of connective tissue and bone matrices [16,17]. Moreover, receptors for androgens have been detected in a high proportion of periodontal and gingival tissue, including fibroblasts from the same source [17,18] which suggests that these tissues may be very sensitive to the anabolic effects of these hormones. 

Androgens contribute to increased bone mass via stimulation of osteoblasts and inhibition of osteoclastic activity. This could be due to a combination of androgen receptor transcriptional activation and non-genomic actions [19], which may be partly dependent on low levels of estrogen. Thus, yields of physiologically active androgen metabolites in cell culture, in this context, may be used as an effective index of anabolic activity in our investigative model.

### 1.3. Oxidative Actions of Nicotine

Smoking is one of the most significant preventable risk factors for periodontal diseases. There is significant documentation of correlations between smoking and the prevalence and severity of periodontal disease [20] resulting from bacterial colonization of oral biofilm and effects mediated on the host response [21]. Nicotine, one of the major components of smoke, may have a damaging effect on the vasculature, humoral and cellular immune system, the inflammatory system and a range of effects throughout the cytokine and adhesion molecule network [22,23,24,25,26]. When ligature- and nicotine-induced periodontitis in rats were investigated using microcomputerized tomography [27], there was significant alveolar bone loss with ligatures, made worse by nicotine, showing damaging effects on alveolar bone microstructure. Gene expression of extracellular matrices and osteoblastic transcription factors were reduced in nicotine treated periodontal ligament cells, with inhibition of mineralized nodule formation [28]. These findings indicate that cell differentiation and mineralization in the periodontium could be affected adversely by nicotine. 

Nicotine-induced damage to gingival fibroblasts could be mediated by modulation of ALP activity [29] in addition to release of free radicals and oxidative actions resulting in impaired wound healing in response to nicotine [30]. It has also been shown in a cell culture study that nicotine significantly depletes glutathione (GSH) levels, which can be overcome by adding GSH to the culture, as demonstrated in PDL fibroblasts [31]. Nicotine, the pharmacologically active and major toxic substance in tobacco, is a significant risk factor for several diseases including lung cancer, cardiovascular and pulmonary disorders. The role of reduced glutathione (GSH) in overcoming nicotine-induced organ toxicity has been reported [32], due to modulation of the biochemical marker enzyme LDH, lipid peroxidation and enhanced antioxidant status. This is a novel finding in this context. 

Both *in vivo* and *in vitro* induction of oxidative stress by nicotine has been reported [33]. Disruption of the mitochondrial respiratory chain with increased generation of superoxide anions and hydrogen peroxide in response to nicotine are some of the mechanisms involved. Vitamin E has been shown to ameliorate nicotine-induced oxidative stress [30,34]. Reduced levels of GSH in serum and neutrophils of smokers have been demonstrated. Further studies with a range of dosing and duration, other antioxidants and free radical scavengers would provide useful information on mechanisms and means for overcoming the oxidative effects of nicotine. Our investigative model using nicotine as a stimulant of oxidative stress is relevant to healing responses in periodontal patients with a history of smoking.

### 1.4. Periosteal Fibroblasts and Osteoblasts

The specialized nature of periosteal tissue comprising fibroblasts, osteoblasts and progenitor cells, provides optimal potential for tissue engineering due to its accessibility and potential for differentiation [35,36,37]; and its osteogenic potential is used extensively for these applications. A population of adherent primary periosteum-derived cells was subjected to both adipogenic and osteogenic culture conditions; and the potential for cell commitment of periosteal cells was compared with those of established fibroblasts and osteoblasts [36]. It is relevant that heterogeneous populations of periosteal cells and fibroblasts are able to express both osteoblast-like and adipocyte-like markers with similar potential. 

This makes us question the progenitor potential of fibroblasts within a multi-lineage tissue such as the periosteum; or whether there are true stem cells within the periosteum. Expanded periosteal cultures may not need enrichment or sorting by molecular markers in order to provide applications for tissue engineering, as indicated in this study. These findings have implications on using periosteal fibroblasts to study responses to EMD in a redox environment potentiated by nicotine in our study, for comparison with osteoblasts. This is relevant to wound healing potential in periodontal lesions.

In the above context, the aim of this investigation is to assess the effect of enamel matrix derivative (EMD) on periosteal fibroblast- and MG63 osteoblast cell cultures and its modulation by nicotine and the anti-oxidant glutathione, by assaying androgen metabolites as biomarkers of oxidative stress relevant to healing responses.

This experimental study could clarify:

(1) The mechanism of action of enamel matrix derivative in periosteal fibroblasts and MG63 osteoblasts.
(2) The modulating outcome of nicotine and glutathione on effects mediated by regenerative agents.

All results are interpreted in the context of redox effects using androgen metabolites as markers of healing and redox potential in cell culture models. The effects of glutathione could have some bearing on counteracting the effects of nicotine during regenerative periodontal treatment amongst smokers.

## 2. Experimental Section

### 2.1. Materials

Authentic steroids from Sigma Chemicals Co., Poole, Dorset, UK were dissolved and redistilled in ethanol (supplied by Merck Chemicals Ltd., Dagenham, Essex, UK) at appropriate concentrations and stored at 4 °C. Radiolabeled androgens (14C-testosterone and 14C-4-androstenedione of specific activity 58 µCi/µmol) were obtained from Amersham International, Amersham, Bucks, UK. The radioactive concentration of 14C-testosterone and 14C-4-androstenedione were 50 µCi/mL toluene and 50 µCi/ 2.5 mL ethanol respectively. The toluene solvent was evaporated to dryness and the radioisotopes were re-dissolved in ethanol for 14C-testosterone.

Ethyl acetate for extraction of metabolites, solvents for thin layer chromatography (benzene/acetone) and chloroform to re-dissolve the dry bulk of extract were all supplied by BDH chemicals (Merck). Thin layer chromatography (TLC) plates were pre-coated silicagel kieselgel 60 (20 cm × 20 cm) and were obtained from BDH Chemicals (Merck) Ltd., Dagenham, Essex, London, UK.

The incubation medium used was Eagle’s Minimum Essential Medium (MEM) with 10% Foetal Bovine Serum (FBS), L-glutamine, antibiotic solution and sodium bicarbonate for titration. The transport medium used for transportation of tissue was Hank’s Balanced Salt Solution (BSS). Tissue culture plastics were provided by Gibco Ltd., Paisley, Scotland. Eagle’s MEM was supplemented with FBS (10% v/v), L-glutamine (200 mM), penicillin (5,000 IU/mL) and streptomycin (5 mg/mL) solution. Nicotine and glutathione were obtained from Sigma Chemicals Ltd., Poole, Dorset, UK. Enamel matrix derivative was obtained from Straumann Ltd., Crawley, West Sussex, UK, in a stock solution of 0.7 mL Emdogain containing 21mg in a hydrophilic carrier of propylene glycol alginate. Suitable dilutions were made in Eagle’s MEM as indicated in the Methods section (page 6, 2.3.1), titrated to a neutral pH with NaHCO_3_.

### 2.2. Periosteal Fibroblasts and MG63 Osteoblasts

The human biopsy materials used in this study were obtained under the guidelines of Kings College London Dental Hospital Trust Ethics Committee. Periosteal tissue was obtained from periodontal patients attending Periodontology, Kings College Dental Institute, London, UK. Tissue was isolated during mucogingival, vertical advancement surgical flap procedures, for correcting gingival recession. The age of patients ranged from 30–50 years. They had all completed non-surgical periodontal treatment comprising root surface debridement prior to surgical procedures. 

Fibroblasts obtained from an inflamed tissue source in males and females exhibit higher metabolic activity with androgen substrates, both at baseline and in response to stimulants, compared with fibroblasts passaged from non-inflamed tissue [38] with insignificant differences between the sexes. Hence the present study samples were not categorized for the genders, based on previous work carried out that showed no difference in testosterone metabolism in chronically inflamed gingival tissue, between males and females; as compared to healthy gingival tissue where testosterone metabolism takes place to a greater extent in male tissue than that of females [38,39]. 

It should be noted that the samples were not pooled, maintaining individual cultures; sample numbers included males and females. Human osteoblasts were derived from a well characterized cell human osteosarcoma cell line referred to as MG63 osetoblasts [40] and gifted by UCL Eastman Dental Institute, London, UK.

The fibroblasts used for all the experiments in the present study were of the 3rd to 9th passage in confluent monolayer culture, in order to ascertain optimal cell function after establishing in culture. Passage numbers of 3–9 have been found to be representative of differentiated cells such as periosteal fibroblasts and MG63 osteoblasts. After the 3rd passage, cells appear to be stable in culture [41]. Metabolic activity in differentiated cells does not appear to be affected by passage number [42], even after extended *in vitro* culturing. 

### 2.3. Experiments

#### 2.3.1. Establishing Optimal Concentrations of EMD, Nicotine and Glutathione

Optimal inhibitory concentrations of nicotine (N) were established using MG63 osteoblasts in monolayer culture, with a range of concentrations (50–500 µg/mL), MEM and radiolabeled 14C-testosterone (T) as substrate, for comparison with controls in the absence of testing agent. The optimal inhibitory concentration of N was established at 200 μg mL for further experiments (N200).

The effect of serial concentrations (1–5 µg/mL) of the antioxidant Glutathione (G) alone was tested previously in MG63 osteoblasts with MEM and radiolabeled 14C-testosterone (T) as substrate, in order to establish the impact it may have per se on androgen metabolism. The optimal effective concentration of G was established at 3 µg/mL (G3). Similar optimal effective concentrations of N and G were established in fibroblast cultures in our previous work [29,43]. The optimal effective concentration of enamel matrix derivative (E) was established using serial concentrations of 15, 30, 45, 60 and 75 µg/mL in MG63 osteoblasts with MEM and radiolabeled 14C-testosterone (T) as substrate. The optimal effective concentration was established at 30 μg/mL for further experiments (E30). Periosteal fibroblasts have functional similarities to MG63 osteoblasts in culture [44], which could be applied for the purpose of this investigation in order to demonstrate effective trends in redox responses; and could be extrapolated to *in vivo* conditions where different cell types co-exist.

#### 2.3.2. Diagnostic Preparation of Multiwells with Optimal Inhibitory Concentrations of Nicotine (N200) and Optimal Effective Concentrations of Glutathione (G3) and EMD (E30).

After establishing optimal concentrations of E, N and G (30, 200 and 3 μg/mL respectively) the experiment was set up using MG63 osteoblasts and periosteal fibroblasts individually with culture medium using radiolabeled 14C-testosterone (T) or 14C-4-androstenedione as substrate, in order to establish the impact of optimal concentrations of E30 and N200 alone and in combinations of N200 + E30, N200 + G3 and ENG for comparison with controls in the absence of agents for yields of DHT (n = 8). 

These two radiolabeled substrates were used for experimental set-ups demonstrating different enzymic pathways leading to the formation of metabolites of interest and provide additional confirmation of the effects of agents tested in periosteal fibroblasts and osteoblasts, using 8 replicates for each experimental set-up. 

#### 2.3.3. Analysis of Metabolites in Cell Culture Eluates from the above Experiments

At the end of a 24 h incubation period, the reaction was terminated and the medium analysed for yields of the steroid metabolite 5α-dihydrotestosterone (DHT), the weaker androgen 4-androstenedione (4-A) and the diols. There were 8 replicates for controls and each of the experimental set-ups. After solvent extraction, the eluate was subjected to TLC in a benzene: acetone solvent system (4:1 v/v); and the separated metabolites were quantified using a radioisotope scanner.

The metabolites were tentatively identified using the mobility of unlabelled standards added to the samples and disclosing them in iodine, showing coincident images with radiolabeled samples using autoradiography, for DHT, 4-androstenedione and the diols. Further confirmation of the identity of steroid metabolites was established by carrying out gas chromatography/mass spectrometry (GC-MS) [43].

### 2.4. Characterization of Steroid Metabolites by gas Chromatography—Mass Spectrometry

As 5α-dihydrotestosterone (DHT) is the most significant biologically active metabolite in stimulating fibroblast matrix synthetic activity, it was characterized as follows. Several incubations were performed with human gingival fibroblasts and unlabelled testosterone (10^−6^ M). After extraction, the identity of DHT as a metabolite in the dried extracts was confirmed by combined capillary gas chromatography—mass spectrometry/g.c-m.s (courtesy of Professor A.I Mallet, St. Thomas’ Hospital, London, UK). The derivatized biological material as the pentafluorobenzyloxime trimethylsilylether (PFBO/TMS) had a molecular ion (557) and mass spectral fragmentation pattern identical to those of authentic PFBO/TMS ether of DHT, but at lower levels due to smaller concentrations of the steroid. Characteristic ions were noted, for example at m/z values of 542 [M-15]+ due to loss of a methyl group; 467 [M-90]+ due to loss of TMS ether; 452 [M-90-15]+ due to loss of TMS ether plus a methyl group and at an m/z value of 360, due to loss of the pentafluorobenzyloxime group. All these procedures have been described in detail [45].

### 2.5. Statistical Methodology

Mean values obtained from the number of cell lines (*n* = 4, *n* = 8) used for individual experiments carried out either as *n* = 4 (for establishing optimal concentrations of agents) or *n* = 8 (for experimental set-ups with the agents) are shown in the Results section. Standard deviations from the mean values are shown in the figures. The cell lines were not pooled and each cell line relates to the subjects. The control incubation in the absence of testing agents served as the comparison for test incubations in the cell lines studied, for each experimental set-up. Data was analyzed statistically using one way ANOVA to test significance of results.

## 3. Results and Discussion

### 3.1. Results

**Figure 1:**
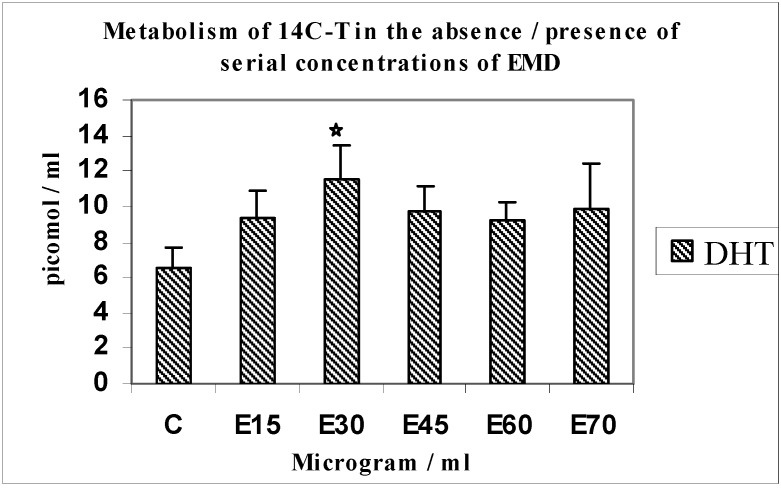
Metabolism of 14C-T in the absence/presence of serial concentrations of EMD.

**Figure 2:**
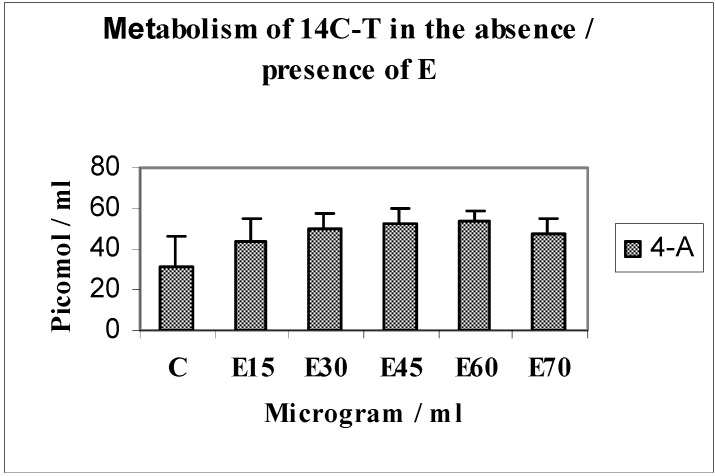
Metabolism of 14C-T in the absence/presence of E.

Mediation of androgen metabolism by serial concentrations of EMD (E15 to 75 µg/mL) used to establish the optimum effective concentration using 14C-T as substrate (Figure 1 and Figure 2).

When monolayer cultures of MG63 osteoblasts were incubated with 14C-T as substrate with serial concentrations of EMD ranging from 15–75 µg/mL, the main metabolites formed in response to EMD were DHT, 4-androstenedione (4-A) or testosterone (T). 

This investigation showed that there was a 1.7-fold increase in yields of DHT (*n* = 4; p < 0.05) in response to EMD at a concentration of 30 µg/mL. An increasing trend was seen for the metabolite 4-A, up to E60 and a slight dip at E70; DHT being the principal physiologically active metabolite, we established that EMD was optimally effective at a concentration of 30 µg/mL; this concentration was used for subsequent experiments. The weaker androgen 4-androstenedione also showed increased yields at E15-70. Points of interest regarding significant differences are indicated with symbols on relevant histograms throughout.

**Figure 3:**
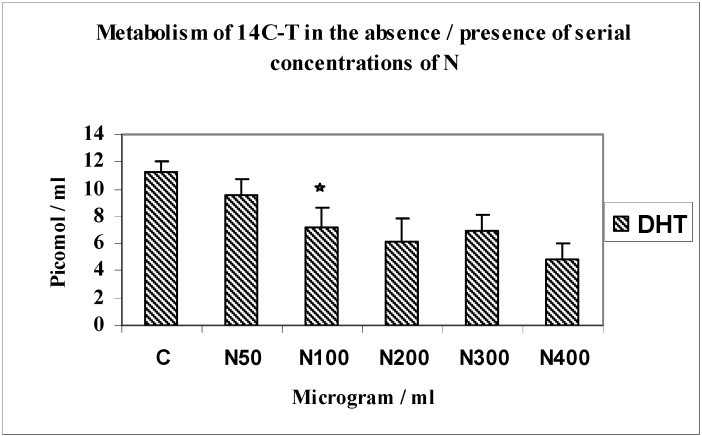
Metabolism of 14C-T in the absence/presence of serial concentrations of N.

Mediation of androgen metabolism by serial concentrations of nicotine (N 50 to 400 µg/mL) used to establish the optimum inhibitory concentration using 14C-T as substrate (Figure 3).

The main metabolites formed by MG63 osteoblasts when incubated with 14C-T as substrate with serial concentrations of nicotine ranging from 50–400 µg/mL were DHT, diols and 4-androstenedione. This investigation showed that there was a 1.8-fold reduction in yields of DHT (*n* = 4; p < 0.0001) in response to nicotine at a concentration of 200 µg/mL. There was not much change in yields of 4-A throughout this experiment. From these results the optimal inhibitory effect of nicotine was established at a concentration of 200 µg/mL. This concentration was used in subsequent experiments.

**Figure 4:**
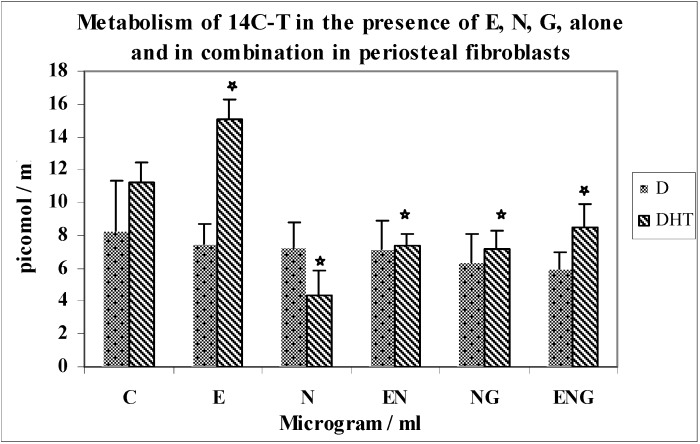
Metabolism of 14C-T in the presence of E,N,G alone and in combination in periosteal fibroblasts.

Effects of optimal concentration of E30, N200 and G3 individually, and in combinations of N200 + G3/N200 + E30/N200 + G3 + E30 on the metabolism of 14C-T in periosteal fibroblasts (Figure 4).

The metabolism of 14C-T by periosteal fibroblasts in response to N, E, G alone and in combination is shown in Figure 4. The formation of DHT was increased by 34% over control in response to EMD (*n* = 8; p < 0.0001).There was a 2.6-fold decrease in DHT synthesis in response to nicotine when compared to controls (*n* = 8; p < 0.0001). The combination of nicotine and E resulted in 70% greater yields of DHT above the value for nicotine alone (*n* = 8; p < 0.0001). Also, the combination of nicotine and G resulted in 63% greater yields of DHT above the value of nicotine alone (*n* = 8; p < 0.0001), which shows that the inhibition of nicotine was overcome by both E and G. The yields of DHT in the incubation with a combination of the three testing agents (E, N, G) showed 97% greater yields than N alone (*n* = 8; p < 0.0001). This is a 15% increase compared to E + N and a 12% increase compared to N + G.

The yields of 4-A showed a similar pattern as those of DHT. There was not much change in the yields of diols in these experiments.

**Figure 5:**
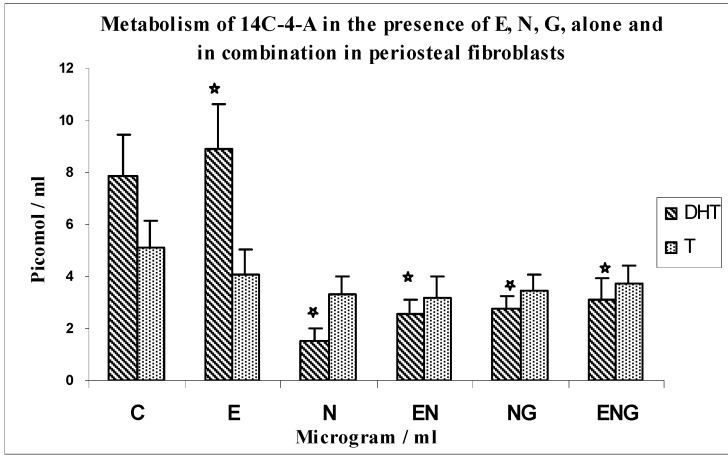
Metabolism of 14C-4-A in the presence of E, N, G, alone and in combination in periosteal fibroblasts.

Effects of optimal concentrations of E30, N200 and G3 individually, and in combinations of N200 + G3/N200 + E30/N200 + G3 + E30 on the metabolism of 14C-4-A in periosteal fibroblasts (Figure 5).

The metabolism of 14C-4-A by periosteal fibroblasts in response to N, E, G alone and in combination is shown in Figure 5. The formation of DHT was increased by 13% over control in response to E (*n* = 8; p < 0.0001). There was a 5.3-fold decrease in DHT synthesis in response to nicotine as compared to controls (*n* = 8; p < 0.0001). The combination of N and E resulted in 73% greater yields of DHT above the value for nicotine alone (*n* = 8; p < 0.0001). Also, the combination of N and G resulted in 86% greater yields of DHT above the value of N alone (*n* = 8; p < 0.0001), which shows that the inhibition of N was overcome by both E and G. The yields of DHT in the incubation with a combination of the three testing agents (E, N, G) showed a 2-fold increase in yields than N alone (*n* = 8; p < 0.0001); a 21% increase compared to E + N (*n* = 8; p < 0.0001) and a 13% increase compared to N + G (*n* = 8; p < 0001).

The formation of T was decreased by 27% over control in response to E. (*n* = 8; p < 0.0008). There was a 55% decrease in yields of T in response to nicotine as compared to controls (*n* = 8; p < 0.0008). The yields of T with a combination of the three testing agents (E, N, G) did not show much difference as compared to N alone, N + G and N + E.

There was not much change in the yields of diols in these experiments.

**Figure 6:**
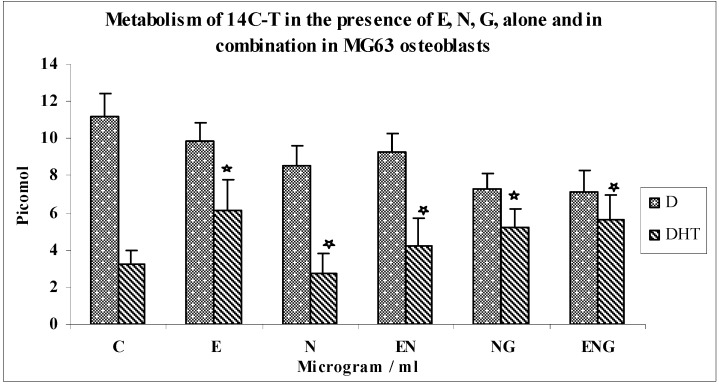
Metabolism of 14C-T in the presence of E, N, G, alone and in combination in MG63 osteoblasts.

**Figure 7:**
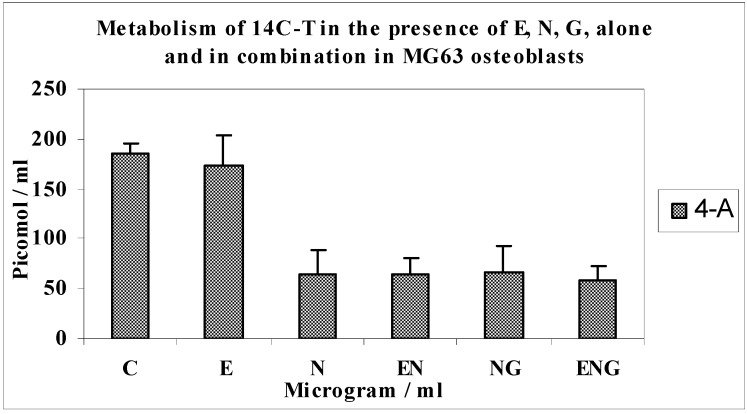
Metabolism of 14C-T in the presence of E, N, G, alone and in combination in MG63 osteoblasts.

Effects of optimal concentrations of E30, N200 and G3 individually, and in combinations of N200 + G3/N200 + E30/N200 + G3 + E30 on the metabolism of 14C-T in MG63 osteoblasts (Figure 6 and Figure 7).

The metabolism of 14C-T by MG63 osteoblasts in response to N, E, G alone and in combination is shown in Figure 6 and Figure 7. The formation of DHT was increased by 88% over controls in response to E (*n* = 8; p < 0.0002). DHT synthesis was decreased by 18% in response to nicotine compared to controls (*n* = 8; p < 0.0002). The combination of nicotine and E resulted in 53% greater yields of DHT above the value for nicotine alone (*n* = 8; p < 0.006). Also, the combination of nicotine and glutathione resulted in 87% greater yields of DHT above the value of nicotine alone (*n* = 8; p < 0.004). 

The yields of DHT in the incubation with a combination of the three testing agents (E, N, G) showed a 2-fold increase in yields than N alone (*n* = 8; p < 0.004). This is a 33% increase compared to E + N (*n* = 8; p < 0.006) and a 9% increase compared to N + G (n = 8, p < 0.004). Similar trends were seen with the metabolite 4-A (Figure 7) to those of DHT. There was not much change in the yields of diols in these experiments.

**Figure 8:**
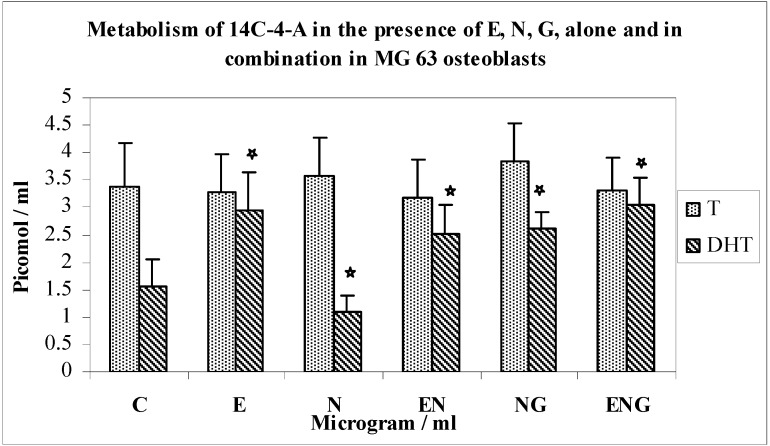
Metabolism of 14C-4-A in the presence of E, N, G, alone and in combination in MG63 osteoblasts.

Effects of optimal concentration of E30, N200 & G3 individually, and in combinations of N200 + G3/N200 + E30/N200 + G3 + E30 on the metabolism of 14C-4-A in MG63 osteoblasts (Figure 8).

The metabolism of 14C-4-A by MG63 osteoblasts in response to N, E, G alone and in combination is shown in Figure 8. The formation of DHT was increased by 85% over controls in response to E (*n* = 8; p < 0.0001). There was 41% decrease in DHT synthesis in response to N as compared to controls (*n* = 8; p < 0.0001). The combination of N and E resulted in a 2.2-fold increase in yields of DHT above the value for nicotine alone (*n* = 8; p < 0.0001). Also, the combination of N and G resulted in a 2.4-fold increase in yields of DHT above the value of N alone (*n* = 8; p < 0.0001), which shows that the inhibition of N was overcome by both E and G. 

The yields of DHT in the incubation with a combination of the three testing agents (E, N, G) showed a 2.7-fold increase in yields when compared with responses to N alone (*n* = 8; p < 0.0001). This was a 20% increase over E + N incubations (*n* = 8; p < 0.0001) and a 15% increase in comparison with N + G incubations (*n* = 8; p < 0.0001). There was not much change in yields of T (Figure 8) and diols (not shown) in these experiments.

**Figure 9:**
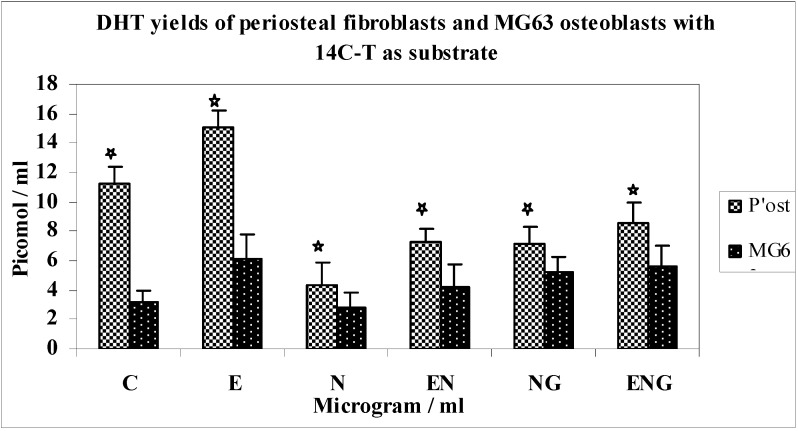
DHT yields of periosteal fibroblasts and MG63 osteoblasts with 14C-T as substrate.

Comparison of yields of DHT between periosteal fibroblasts and MG63 osteoblasts using 14C-T as a substrate (Figure 9).

The metabolism of 14C-T in periosteal fibroblasts (HPF) produced baseline control yields of DHT approximately 3 times greater than that of MG63 osteoblasts. In response to E the increase in yield of DHT was 35% greater for HPF than MG63 osteoblasts. Nicotine caused a 13-fold greater decrease in yields of DHT in HPF than MG63 osteoblasts.

When E was added to N, the increase in yields of DHT was 2-fold greater for HPF than for MG63 osteoblasts. There was a 14% increase in yield of DHT in HPF compared to MG63 when G was added to N. When E + G + N were combined, HPF produced 46% greater yields of DHT when compared with MG63 osteoblasts.

**Figure 10:**
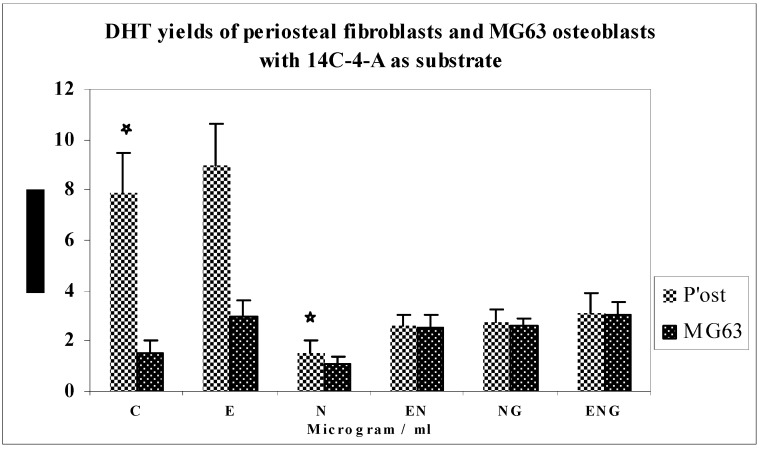
DHT yields of periosteal fibroblasts and MG63 osteoblasts with 14C-4-A as substrate.

Comparison of yields of DHT between periosteal fibroblasts and MG63 osteoblasts using 14C-4-A as a substrate (Figure 10).

The metabolism of 14C-4-A by periosteal fibroblasts produced baseline control yields of DHT approximately 5 times greater than that of MG63 osteoblasts. The increase in yield of DHT in response to E was the same for both cell types. The decrease in yields of DHT was 14 times greater for HPF than MG63 osteoblasts in response to nicotine. There was not much difference in the yield of DHT between both cell types in response to E + N, G + N and E + N + G. 

### 3.2. Discussion

The results of this investigation demonstrated that human periosteal fibroblasts and osteoblasts metabolized 14C-T mainly to 5α-dihydrotestosterone (DHT), diols (D) and 4-androstenedione (4-A), while 14C-4-A was mainly metabolized to DHT and testosterone (T), both with controls and testing agents in accordance with enzymic pathways. 14C-4-A as a substrate is first metabolized to small amounts of testosterone and subsequently reduced to DHT by 5α-reductase [29,46]. Using these two substrates reinforces the implications of yields of metabolites in response to the testing agents.

Testosterone, the main male hormone binds androgen receptor (AR) both directly and indirectly via conversion to DHT catalyzed by the enzyme 5α-reductase. DHT is considered to be the most potent natural AR natural ligand [47] with a 2-10-fold greater potency than testosterone in androgen-responsive tissues. It has greater affinity for receptor binding [13]. Testosterone can be irreversibly converted to DHT by 5α-reductase. DHT seems to exert anti-apoptotic effects. Steroid hormones activate signal transducers that trigger a variety of cellular responses. DHT prevents H_2_O_2_—induced apoptotic cell death by activation of catalase, downregulation of p38 MAPK, JNK/SAPK, and NF-kappaB via the androgen receptor. These effects are blocked by flutamide treatment which confirms mediation of these actions via androgen receptor [48] and the efficacy of DHT as a marker of oxidative stress. 

The testing agents used were EMD (E), glutathione, and nicotine in an experimental model which can be extrapolated to the regenerative capacity of EMD in smokers simulating an oxidative stress-inducing environment and its alleviation by antioxidants. These agents were tested either separately or in combination in order to determine individual and combined effects. 

The anabolic potential of EMD is well-documented [49,50,51,52] and used extensively in regenerative periodontal treatment. When EMD was used alone at concentrations ranging from 15 to 75 µg/mL it stimulated DHT synthesis in osteoblasts with 14C-T as substrate. There was a 1.7-fold increase in yields of DHT in response to EMD at a concentration of 30µg/ml indicating increased anabolic activity in cultured osteoblasts, with similar trends seen in the yields of weaker metabolites such as 4-A. It was previously reported that EMD may significantly enhance ALP activity, an enzyme associated with turnover of connective tissue and bone, in periodontal ligament fibroblasts [8,53] and MG63 osteoblasts. This could partly explain the increase in yields of DHT, observed in this investigation.

Biological data regarding EMD at the cellular and molecular levels have been evaluated in the context of periodontal wound healing and tissue formation [1] and support their actions in promoting new periodontal tissue formation and wound healing, in an animal experimentation model [54], and humans [52,55]. Aspects of clinical and scientific rationale for the applications of EMD have been documented [56]. 

When nicotine was used alone in concentrations ranging from 50–400 µg/mL with 14C-T as substrate, the yields of DHT were significantly inhibited at 200 µg/mL. There was a 1.8-fold reduction in yields of DHT in response to nicotine at a concentration of 200 µg/mL indicating decreased anabolic activity in cultured MG63 osteoblasts. There was not much change in the yields of weaker metabolites such as 4-A and T.

It has been proposed that free radicals and oxidants may mediate some of the damaging effects of nicotine [30] and impaired wound healing. It has also been reported that nicotine can inhibit the formation of matrix-stimulatory steroid metabolites in fibroblasts, partly due to inhibition of ALP activity suggestive of nicotine-induced damage mediated by this mechanism [29]. This appears to be in agreement with our investigation related to MG63 osteoblasts whereby the generation of free radicals and superoxide in the presence of nicotine, could trigger enzymes mediating the metabolism of androgen substrates; thereby contributing to reduced yields of metabolites. Reduced yields of the androgen metabolite, DHT, observed in this investigation may have possible implications on healing in the smoking population.

Nicotine has been identified as a significant risk factor for several diseases being a major toxic component of cigarette smoke. The role of reduced glutathione (GSH) in nicotine-induced organ toxicity [32] has shown that the protective effect of GSH was exerted by modulation of the biochemical marker enzyme LDH, lipid peroxidation and augmentation of the antioxidant defence system. Documented literature suggests a redox imbalance in smokers which may be an important factor in mediating tissue damage in several tissues and that antioxidants are effective in overcoming the damaging effects of nicotine [57]. The actions of nicotine closely identify with responses induced by an oxidative biomarker [58]. In this study, we used glutathione, present in mammalian cells, as an antioxidant as it has long been recognized in essential defense and many other cellular functions in most cells; such as acting as a redox buffer to preserve the reduced intracellular environment [59,60].

We found that the inhibitory effect of nicotine on the metabolism of androgen substrates to the bioactive redox marker DHT, was overcome by enamel matrix derivative in both HPF and MG63 osteblasts; and in both substrates. There was a significant increase in yields of DHT when the cells were incubated with EMD. The oxidative stress caused by nicotine could interfere with function of both cell types by increasing the formation of free radicals and disrupting the natural antioxidant defence mechanism. It is relevant that in combination with EMD, the detrimental effect of nicotine on the yields of androgen biomarkers was overcome; as it implies an antioxidant effect exerted by EMD. The anabolic effects of androgen on bone and connective tissue turnover could be compromised in a smoking population via these mechanisms.

Also, when glutathione was added to both HPF and MG63 osteoblasts in both substrates, it seems to have a significant stimulatory influence on metabolic yields of DHT and was able to overcome the inhibitory effect caused by nicotine. This finding demonstrates the harmful oxidant nature of nicotine in inflammatory diseases and the importance of the antioxidant glutathione in overcoming its inhibitory effect. Exposure to nicotine has been shown to induce early stress response mRNA expression and significant depletion of intracellular glutathione levels in a dose dependent manner, which was overcome by adding it to the culture [61]. Results of our investigation showed that detrimental effects of nicotine were overcome by glutathione suggesting that the effects of nicotine on androgen metabolism could be partly exerted through an oxidative pathway. Normal function of fibroblasts and osteoblasts is critical for maintenance of periodontal health and for optimal wound healing. Factors that inhibit functions of such cells may also impair tissue repair and regeneration; enhancement of their function would be of great clinical value amongst smokers with impaired and defective wound healing. 

We extended our investigation to look at the effects of combined application of E and glutathione on androgen metabolism in both cell types with nicotine (N + E + G). It seemed to have a moderate increase in metabolic yields of DHT as compared to E or glutathione alone. This implies enhanced antioxidant and matrix stimulatory activity which has useful applications in adjunctive regenerative therapy using EMD (E).

For other weaker metabolites, the trend shown was either the same as those for DHT or not much change. In some cases there was an inverse relationship. This may be attributed to compensatory enzymic pathways whereby an increase in weaker metabolites overcomes reduced yields of the physiologically active metabolite.

With regard to differences in yields of DHT between periosteal fibroblasts and MG63 osteoblasts using both substrates, it was shown that HPF produced baseline control yields of DHT approximately 3 times greater than that of MG63 osteoblasts with 14C-T, and approximately 5 times greater baseline yields with 14C-4-A as substrate. Also, there was a dramatic decrease in yields of DHT in HPF cultures with both substrates in response to nicotine when compared with MG63 osteoblasts. The response of HPF to subsequent incubations with EMD and glutathione alone or in their combinations also showed higher yields of DHT than with MG63 osteoblasts, implying variations in their synthetic capacity. This could be due to different target tissue actions of androgens [62] or the high number of androgen receptors detected in a high proportion of fibroblasts [18].

Analysis of combined roles of EMD, glutathione and oxidative stress will be required to fully understand how they may contribute to healing responses amongst smokers. An *in vitro* investigation has advantages in the ability to explore temporal effects of EMD on cell functions, gene expression and regulation of protein in the presence or absence of different factors. Molecules with potent pro-glutathione effects have been reviewed and they show great promise as therapeutic agents to treat different pathologies associated with chronic inflammation and oxidative stress [60], reinforcing our findings. These changes are not easily detected *in vivo* models and results of our investigation may provide useful evidence to support the initiation of clinical trials. This has implications for a novel therapeutic approach to counteract the effects of nicotine during regenerative periodontal treatment amongst smokers. 

## 4. Conclusions

Nicotine exerted oxidant effects on androgen metabolism as shown by significant inhibitory effects on yields of the androgen biomarker DHT, in both HPF and MG63 osteoblasts, which were overcome by EMD and the antioxidant glutathione. This is suggestive of a possible oxidative effect of nicotine on both cell types. It affected yields of androgen metabolites functioning as biomarkers of redox status and repair, with potential implications on healing in an oxidative stress-inducing environment.

EMD and glutathione alone and in combination were capable of overcoming the effects of nicotine. The results of our investigation are reinforced by the antioxidant effects of androgens in activating relevant genes [63] and their direct antioxidant capacity [64] in making them effective biomarkers of healing and redox interactions in the context of this study. Critical stages of wound healing are inhibited in an inflammatory environment [65]; which may be attenuated by EMD, in addition to other regenerative agents [66]. In the context of our study, these mechanisms open interesting possibilities for the applications of regenerative agents in an inflammatory redox environment, characteristic of the periodontal bone lesion. 
